# A Case of Chronic Abdominal Neuropathic Pain and Burning after Female Genital Cutting

**DOI:** 10.1155/2015/906309

**Published:** 2015-06-02

**Authors:** Vicky Hadid, Michael Haim Dahan

**Affiliations:** Obstetrics and Gynecology, Royal Victoria Hospital, McGill University Health Centre, 687 Pine Avenue West, Montreal, QC, Canada H3A 1A1

## Abstract

*Introduction*. Female genital cutting is prevalent in the Middle Eastern and African countries. This ritual entails not only immediate complications such as infection, pain, and haemorrhage, but also chronic ones including dysmenorrhea and dyspareunia. However, there is limited data on neuropathic pain secondary to female genital mutilation when searching the literature. *Case*. This case discusses a 38-year-old female with a history of infibulation who presented with a chronic burning abdominal and anterior vulvar pain including the related investigations and treatment. *Discussion*. This case brings to light the additional delayed complication of this ritual: sensory neuropathy. Our goal is to educate health professionals to be aware of these complications and to appropriately investigate and treat them in order to find a solution to relieve the patients' symptoms.

## 1. Introduction

Although the practice of female circumcision is illegal in westernized countries, there exist many women who have endured this procedure. This practice most commonly occurs in Africa and the Middle East, and estimates are that over 100 million women have been affected [1]. Female genital mutilation is defined as any procedure that intentionally alters or causes injury to the female genital organs for nonmedical reasons. It can be classified into four major types: clitoridectomy, excision, infibulation, and other forms of genital injury [1, 2]. Although this ritual is thought to be beneficial by the communities who still practice it, these practices entail a multitude of medical complications for the female, both acute and chronic. The most commonly cited long-term complications are dysmenorrhea, dyspareunia, and chronic vaginal infections [2]. Other complications include voiding dysfunction, scarring, and higher rates of infertility [1, 2].

The vulva contains many anatomic structures from blood supply to nerve innervation. The latter includes the pudendal, ilioinguinal, and genitofemoral nerves which are essential for sensory function [3, 4]. Due to this rich network of innervation, it is strange that there exists limited data on neuropathic pain secondary to female genital mutilation. The breadth of the literature focuses on dyspareunia or dysmenorrhea. Interestingly there have only been two cases reporting the formation of a clitoral neuroma secondary to excision and scarring [5, 6].

This case report will discuss the delayed neuropathic complications that may occur in a woman who experienced female circumcision without evidence of any neuromas. Our goal is to educate health professionals to be aware of these complications in addition to their investigations and treatment in order to find a solution to relieve the patients' symptoms.

## 2. Case

This case discusses a 38-year-old female, gravida 5, para 2, aborta 3 (spontaneous abortions), originally from Cote d'Ivoire, who presented with severe unremitting chronic pelvic pain, which had failed to be addressed by three physicians. She had undergone an infibulation between the ages of 7 and 8 years. It was encouraged by her parents who felt that to marry off a daughter who had not had an infibulation would bring disgrace to the family. Her obstetrical history includes two uneventful spontaneous vaginal deliveries at term in her country of origin in 2003 and 2005. The patient did not recollect any significant lacerations, bleeding, blood transfusions, or infections but did mention that her introitus was cut open during labor.

Upon initial assessment, she complained of a 5-year history of pelvic burning. It was described as painful burning that involved the lower half of the abdomen and anterior half of the vulva radiating out to the thighs. The left side had worse pain than the right and quantified as 8 out of 10 in severity. There were no aggravating or ameliorating factors. The pain was so significant that it made daily function and sexual intercourse impossible. There were no other neurological deficits or back pain upon further questioning. She reports regular menstrual cycles every 26–29 days, accompanied with dysmenorrhea. She first had intercourse at age 18. Repair of the infibulations was performed three years before, when she immigrated to North America, which required three surgical procedures and a skin graft for which we were unable to obtain an operative record.

As part of her physical exam, the vulva was examined and it was quite tender to touch in the anterior areas. She stated this tenderness had improved after her surgeries to correct the infibulation but persisted. No erythema was noted or any vestibular tenderness even after the *Q*-tip test. No abdominal or pelvic masses were present. No visible or palpable neuromas were present. There was no vaginal discharge or odour. The glans of the clitoris, a part of the clitoral body and hood, as well as labia minora were completely removed. Chlamydia and gonorrhoea polymerase chain reaction testing as well as a urine culture were negative. A pelvic ultrasound demonstrated a small posterior intramural fibroid, less than 1 cm in diameter, not distorting the cavity.

Based on these neurologic symptoms with no evidence of a physical pathology, a trial of nortriptyline was commenced, titrating the dose weekly from 10 mg to 50 mg. Furthermore, a neurologist was consulted; however, no additional testing was recommended as it was deemed not to be beneficial.

Three months later, she returned to clinic reporting that her symptoms had subsided to the point that she could have regular intercourse. Therefore, she was continued on nortriptyline 25 mg bid. However, one month later, in February 2014, she presented to the clinic complaining that the burning sensation had returned despite compliance with the medication. Again, she describes it as “hot and burning” and like a diffuse ulcer. She was asked to return again in 2 weeks while continuing the medication. Once more, she complained that the burning sensation was persistent; therefore, nortriptyline was stopped and gabapentin 100 mg tid started. This resulted in resolution of the pain. Once pain-free, her sexual function normalized.

## 3. Discussion

This case brings to light the additional delayed complication of genital cutting: sensory neuropathy. The differential diagnosis of a patient who presents with burning vulvar pain would include infectious causes such as vaginitis, urinary tract infection, vestibulitis, dermatitis, abuse, and finally neuropathy. Our initial infectious workup returned negative and the pelvic exam was unremarkable, which effectively rules out the infectious or dermatological causes. Additional neurologic workup is not warranted as there are no tests to document pudendal neurologic hyperactivity.

Her history of female genital mutilation cannot be ignored as this is likely the root cause of her problems. Aside from the mental trauma this procedure can cause, it is possible for a nerve to have been injured or entrapped in scar tissue, thus causing her symptoms. Nerve entrapment syndrome can occur many years after surgery [7]. Sensation to the female vulva and perineum is supplied mostly via the pudendal nerve and ilioinguinal nerve. The pudendal nerve (S2–4), which has both motor and sensory functions, exits Alcock's canal and divides into three major branches: the dorsal nerve of clitoris, the inferior anal/rectal nerve, and the perineal nerve which gives off the posterior labial nerves. The anterior labial nerve branches from the ilioinguinal nerve [3, 4]. Damage to any one of these nerves may result in a neuropathy. A neuropathy is defined as any disorder of the nervous system [8]. More specifically, her symptoms suggest a peripheral neuropathy which was likely caused by entrapment with referred symptomatology [7, 9].

The closest recognised diagnosis to this symptomatology is pudendal neuralgia with a reported incidence ranging from 1 in 100 to 1 in 100 000 [10]. It is defined as a burning neuropathic pain in the distribution of the pudendal nerve as described earlier [11]. Additionally there are a few cases of pudendal neuralgia where patients may have pain outside the innervated region of the pudendal nerve including lower abdomen, thigh, and back as in the case described [12]. However, our patient did not report pain exclusively when sitting which is a component of the diagnostic criteria for pudendal neuralgia [12]. Another term used is pudendal nerve entrapment. This may occur in women with surgical injury, described in pelvic reconstruction surgeries including sacrospinous ligament fixation, pelvic trauma, and childbirth [7, 10, 13]. Additionally, myofascial pelvic pain syndrome, described as short, tight, and tender pelvic floor muscles with hypersensitive trigger points, may be contributing to her symptoms, although on examination no taut band of muscle was palpated [14].

This patient had infibulation, which is the more severe form of female genital mutilation. This process involves narrowing of the vaginal opening by cutting the inner and outer labia, with or without removal of the clitoris (type 3 WHO) [1]. Since the branches of the pudendal nerve terminate in the region where excision and suturing are performed, it is possible that these nerves are injured during the process, thus leading to neuropathic pain. In this case, the patients' symptoms presented in a delayed fashion. This can be attributed to the nerve trauma that may have occurred during childbirth. Due to the narrowing of the vaginal space secondary to the infibulation, when a woman labors, there may be greater compressive forces/tension on these nerves, thus leading to the delayed symptomatology. Similarly, it is well known that there are greater obstetrical risks in women who have experienced infibulation including a prolonged second stage of labor, increased rates of perineal tears and episiotomies, and postpartum hemorrhage [15]. Although she denied any complications with her deliveries, we can only speculate that these factors may have contributed to her symptomatology, in addition to the unsafe delivery conditions.

Unfortunately, the treatment options, both medical and surgical, are limited in this population. Ideally, the nerve should be released if it had been trapped. However, since these procedures are more likely to go unrecognized for many years, a delayed reversal is unlikely to reduce the chances of neuropathic pain. Other options include medical treatments with topical lidocaine, opioids, or antidepressants/anticonvulsants used for the management of chronic pain [16]. In our case, the initial treatment was the tricyclic antidepressant, nortriptyline, which was successful for a limited time. Once the symptoms returned she was switched to gabapentin, an anticonvulsant. Both these medications are considered first-line agents in treating chronic pain according to guidelines endorsed by the American Pain Society [16, 17]. Another first-line treatment would include Serotonin-Norepinephrine reuptake inhibitors (SNRIs), which are considered dual action antidepressants since they block presynaptic serotonin and norepinephrine transporter proteins [17, 18]. A SNRI will be our next choice of treatment if gabapentin proves to fail. Additionally, a referral to a sex therapist for psychosexual care is an important step in the recovery process of a patient who has suffered so extensively. However, in our case, once she was pain-free, she reported normal sexual function.

It should be noted that the onset of the pain predated the repair of the infibulation by two years. Although the repair of the infibulation did improve the pain slightly, it remained chronic and debilitating. In theory, any surgical procedure entails risks which can include neurological ones. Therefore, the repair of the infibulations could cause this type of neurologic pain to occur.

This patient had seen three gynaecologists seeking care for this pain. All failed to develop a working hypothesis towards symptomatology or more importantly a solution for pain appeasement. No masses or signs of a neuroma were present on exam. Since this is the first case to describe this pain in the literature, her pain went unabated because the possibility of the pathology did not occur to the prior physicians that were consulted. The last physician to see her realized that most likely nerve entrapment was present and successfully implemented a chronic pain protocol. Most likely this type of pain is underdescribed in these patients for the following reasons: patients who have endured female genital cutting are not forthcoming regarding their symptomatology and healthcare providers may incorrectly describe it as dysmenorrhea or dyspareunia. Upon questioning, the patient stated that many women in her community experience similar chronic pain/burning but that they are too embarrassed to come forward, suggesting that this complication is more common than the medical literature documents.

## 4. Conclusion

In conclusion, female genital cutting can have debilitating short- and long-term effects including delayed neuropathy as seen in this case. Currently, the treatment of chronic neuropathic pain is limited with varying individual response rates. As healthcare professionals, our duty is to recognize and support these women affected by female genital mutilation, both psychologically and medically.

## References

[1] World Health Organization (2008). *Eliminating Female Genital Mutilation: An Interagency Statement*.

[2] Nour N. M. (2013). *Female Genital Cutting*.

[3] Netter F. H. (2006). *Atlas of Human Anatomy*.

[4] McCrory P., Bell S. (1999). Nerve entrapment syndromes as a cause of pain in the hip, groin and buttock. *Sports Medicine*.

[5] Fernández-Aguilar S., Noël J.-C. (2003). Neuroma of the clitoris after female genital cutting. *Obstetrics and Gynecology*.

[6] Abdulcadir J., Pusztaszeri M., Vilarino R., Dubuisson J.-B., Vlastos A. T. (2012). Clitoral neuroma after female genital mutilation/cutting: a rare but possible event. *Journal of Sexual Medicine*.

[7] Gray J. E. http://www.uptodate.com/contents/nerve-injury-associated-with-pelvic-surgery?source=search_result&search=Nerve+injury+associated+with+pelvic+surgery&selectedTitle=1%7E150.

[8] Rutkove S. B. (2014). *Overview of Polyneuropathy*.

[9] Simionescu L. (2014). *Traumatic Mononeuropathies*.

[10] Hibner M., Desai N., Robertson L. J., Nour M. (2010). Pudendal neuralgia. *Journal of Minimally Invasive Gynecology*.

[11] Robert R., Prat-Pradal D., Labat J. J. (1998). Anatomic basis of chronic perineal pain: role of the pudendal nerve. *Surgical and Radiologic Anatomy*.

[12] Labat J.-J., Riant T., Robert R., Amarenco G., Lefaucheur J.-P., Rigaud J. (2008). Diagnostic criteria for pudendal neuralgia by pudendal nerve entrapment (Nantes Criteria). *Neurourology and Urodynamics*.

[13] Lien K.-C., Morgan D. M., Delancey J. O. L., Ashton-Miller J. A. (2005). Pudendal nerve stretch during vaginal birth: a 3D computer simulation. *The American Journal of Obstetrics and Gynecology*.

[14] Elkadry E., Mynihan L. K. (2015). *Clinical Manifestations and Diagnosis of Myofascial Pelvic Pain Syndrome in Women*.

[15] De Silva S. (1989). Obstetric sequelae of female circumcision. *European Journal of Obstetrics Gynecology and Reproductive Biology*.

[16] Rosenquist E. W. K. (2014). *Overview of the Treatment of Chronic Pain*.

[17] Dworkin R. H., O'Connor A. B., Backonja M. (2007). Pharmacologic management of neuropathic pain: evidence-based recommendations. *Pain*.

[18] Katzung B. G., Masters S. B., Trevor A. J. (2009). Antidepressant agents. *Basic and Clinical Pharmacology*.

